# Coital Frequency and the Probability of Pregnancy in Couples Trying to Conceive Their First Child: A Prospective Cohort Study in Japan

**DOI:** 10.3390/ijerph17144985

**Published:** 2020-07-10

**Authors:** Shoko Konishi, Tomoko T. Saotome, Keiko Shimizu, Mari S. Oba, Kathleen A. O’Connor

**Affiliations:** 1 Department of Human Ecology, School of International Health, Graduate School of Medicine, The University of Tokyo, Tokyo 113-0033, Japan; 2 Department of Anthropology, University of Washington, Seattle, WA 98195, USA; oconnork@uw.edu; 3 Interdisciplinary Advanced Medical Research Laboratory, Louis Pasteur Center for Medical Research, Kyoto 606-8225, Japan; kongo.tomow@gmail.com; 4 Department of Zoology, Faculty of Science, Okayama University of Science, Okayama 700-0005, Japan; shimizu@zool.ous.ac.jp; 5 Department of Medical Statistics, Faculty of Medicine, Toho University, Tokyo 143-8540, Japan; mari.oba@med.toho-u.ac.jp

**Keywords:** antimüllerian hormone, Asia, fertility, hCG test, infertility, LH test, sexual behavior, time-to-pregnancy, urinary estrogen, urinary progesterone

## Abstract

*Background*: Low fertility persists but remains unexplained in Japan. We examined whether the probability of pregnancy was influenced by coital frequency, age, reproductive age (assessed by antimüllerian hormone, AMH), and BMI. *Methods:* We established a two-year prospective study with a sample of hormonally monitored Japanese women aged 23–34 years wanting to conceive their first child. For a maximum of 24 weeks participants recorded menstrual bleeding, sexual intercourse, ovulation, and pregnancy. Additional information on pregnancy and infertility treatment was collected one and two years after intake. *Results:* The natural conception rate and coital frequency were both low in this sample. Among 80 participants, 44% (35) naturally conceived in 24 weeks. After two years, 74% (59) of women had delivered or were currently pregnant, 50% (40) due to natural and 24% (19) due to assisted conception, and 5% (4) were lost to follow-up. By two years, 56% (45) of women had sought fertility treatment. In 18% (58/319) of the observed ovarian cycles across 24 weeks there was no intercourse in a fertile period. Higher coital frequency at intake was associated with increased probability of conception by 24 weeks of follow-up (OR 1.23, 95%CI 1.02, 1.47). Chronological age, reproductive age, and BMI were not associated with the probability of pregnancy at 24 weeks. *Conclusions:* Our results suggest that first, natural conception rates could potentially increase with more frequent and well timed intercourse, and second that further work is needed to understand why even in a motivated sample of women monitoring their fertile periods, both the conception and coitus rates were low.

## 1. Introduction

Low fertility has persisted in Japan for the last several decades. The total fertility rate (TFR) of Japan was 1.4 in 2019 [1]. The National Fertility Survey of Japan in 2015 [2] reported that among married couples with no children, the proportion who had ever received infertility treatment or examination was 17.8% for couples with wives aged 20–29 years, 32.8% for those aged 30–39 years, and 29.4% for those aged 40–49 years. These figures suggest the frequent use of assisted reproductive technologies among couples trying to conceive. In fact, Japan had the highest number of follicular aspirations of approximately 60 countries participating in the International Committee for Monitoring Assisted Reproductive Technology in 2008–2010 [3]. 

The Japan Society of Obstetrics and Gynecology defines infertility as a couple’s inability to conceive after 12 months of unprotected sexual intercourse [4]. According to this definition, a couple experiencing times to pregnancy (TTP) longer than 12 months is judged to be infertile. Our previous study [5] targeting women in Japan estimated that the proportion of couples with TTP longer than 12 months was higher by approximately 10 points compared to similar estimates based on women in England and Scotland [6], even after adjusting for female age.

The factors contributing to low fertility, and the high rate of infertility, in Japan remain unclear. Among the Japanese population, the overall TTP may be increasing secularly, considering first that the mean age at marriage and childbearing is increasing [2] and second, that TTP tends to be longer as men and women age, reflecting reduced fecundability (the probability of pregnancy per month or ovarian cycle) [5,6,7,8]. Therefore, reproductive aging may contribute to the lower probability of pregnancy of couples trying to conceive and to an increase in infertility. Aging of both females and males affects fecundability [9,10,11,12]. Even at the same chronological age, the degree of reproductive aging varies across individuals [13]. Therefore, in addition to chronological age as a biological marker, other biomarkers are used as indicators of reproductive age—such as follicle stimulating hormone, antimüllerian hormone (AMH), and inhibin B, which have been used to define stages of reproductive aging [14]. AMH is one of the earliest indicators of reproductive ageing [14,15]. Although AMH is not an established biomarker for spontaneous conception [16], it is an indicator of ovarian reserve, and it does not vary across the ovarian cycle, making it a useful measure of reproductive aging and fertility potential [14,17,18]. We examined both chronological age and reproductive age (as measured by AMH) as biological determinants of TTP in a Japanese sample of reproductive aged women.

Body mass and weight have also been associated with TTP, with both overweight and underweight being associated with longer TTP. A high or low BMI is associated with longer or shorter TTP via various types of altered hormonal and cyclical events which prevent fertile cycles [19]. In contrast to most Western settings, a substantial number of Japanese women are of low weight and BMI. The 2015 National Nutrition Survey revealed that 22.3% and 15.5% of Japanese women aged 20–29 and 30–39 years, respectively, were underweight, defined as BMI < 18.5 kg/m^2^ [20]. Previous epidemiological studies from other countries have reported associations between underweight and longer TTP [21,22,23]. Additionally, more recent studies have reported adverse effects of being underweight on the success rate of in vitro fertilization [24,25]. Given the high prevalence of underweight in young reproductive age women, the question arises as to whether this might contribute to low fertility in Japan. We hypothesized that lower weight would be associated with a lower probability of pregnancy.

The probability of conception in a given fertile cycle is also influenced by behavioral factors. One key proximate determinant of natural fertility [26] (pp. 67–73)—the frequency of intercourse in the fertile period—has a direct and strong influence on the probability of pregnancy and thus on TTP. The frequency and timing of sexual intercourse can be controlled by couples but is rarely included in studies examining TTP or low fertility. In a recent study with a nationally representative sample, a lower frequency of marital intercourse was found in Japanese couples compared to couples in Western settings [27], suggesting one possible explanation for a longer TTP in Japanese couples. Thus, the current study aimed to further investigate frequency of intercourse and probability of natural conception. 

The objective of the present investigation was to focus on chronological age, reproductive age, body weight, and frequency of intercourse, and test these in association with a prospectively monitored outcome: the probability of natural conception within 24 weeks. These determinants are associated with fecundability (probability of pregnancy) and TTP in Western settings [6,7,9,10,11,21,22,23,28,29] but are largely unknown with respect to Japanese fertility. However, they are of interest as contributors to low fertility in Japan, given current behavioral, epidemiologic, and demographic trends. 

## 2. Materials and Methods 

The Baby Machi (meaning “waiting for a baby” in Japanese) study was a prospective cohort study targeting couples who planned to conceive their first child. The participants (*n* = 80) were followed for a maximum of 24 weeks or until pregnancy was clinically confirmed. Additional follow-up questionnaires were sent one and two years after the enrolment to collect information on infertility treatment and reproductive outcomes. This report examines a main outcome of natural (spontaneous) conception within 24 weeks of follow-up.

This study was approved by the Ethics Committee of the Graduate School of Medicine, the University of Tokyo (10878-(2)). Participants provided signed informed consent upon participation.

### 2.1. Eligibility Criteria 

Eligible women were aged 20 to 34 years, either married or engaged, were not currently pregnant, wanted to become pregnant, and were off contraceptives or planning to discontinue contraception within a month. To be eligible, they had not used hormonal contraception or an intrauterine device (IUD) during the past three months. Eligible women were also nulliparous; never diagnosed with recurrent miscarriage, endometriosis, uterine fibroid, or polycystic ovary syndrome (PCOS); never had uterus or ovary removal and had never been advised by a medical doctor to avoid pregnancy due to a disease. Women whose partners were sterilized or had a testicle removed were not eligible. Couples who had ever consulted a doctor for infertility treatment or examination or who planned to consult with a doctor for infertility treatment within the next six months were not eligible. All eligible women had menstrual bleeding more than twice within the past three months. Detailed information about methodology is described in Supplemental Information.

### 2.2. Study Procedure

After enrolling in the study online, the women were emailed a baseline questionnaire about their reproductive history and lifestyle characteristics. A total of 80 women participated in the study intake at the University of Tokyo (Hongo campus) on 15 November 2015. Participants were given an explanation of the study purpose and procedure and signed an informed consent form. The participants had venous blood collected and height and weight measured. Blood specimens were centrifuged, and serum specimens were stored at −80 °C until laboratory analysis.

During 24-weeks of follow-up, participants used urinary ovulation and pregnancy test kits, following instructions, to monitor their reproductive status. They used luteinizing hormone (LH)-based ovulation test kits (Dotest LH, ROHTO Pharmaceutical Co., Ltd., Osaka, Japan) starting from 17 days before the expected starting day of the next menstrual bleed and continued use for seven consecutive days. The participants were instructed to use human chorionic gonadotropin (hCG)-based pregnancy tests on whichever came first within their menstrual cycle, (1) the first day of menstrual bleed, or (2) the expected starting day of the next menstrual bleed. An additional pregnancy test was conducted seven days after the first test in each cycle, thus two pregnancy tests were completed in every cycle. 

The ability of the urine LH kits to estimate day of ovulation and thus the fertile period of an ovarian cycle was evaluated by comparison to an estrogen to progesterone ratio indicating day of luteal transition. A subsample of study women (*n* = 18) collected daily urine specimens for up to 24 weeks that were assayed for metabolites of estrogen and progesterone. See supplemental material for details. 

Participants in the main study recorded daily information on the test results (hCG and LH), menstrual bleeding, and sexual intercourse, and reported these results online once a week. When a positive pregnancy test was reported, an additional questionnaire was sent to collect information on the pregnancy outcome. If an ongoing pregnancy was confirmed by a medical doctor, then weekly participation was discontinued, with pregnancy outcome assessed at 1 and 2 year follow up. If termination of pregnancy (abortion) was reported in the questionnaire, then the participants were asked whether they wanted to finish or continue follow-up. Participants emailed the principal investigator (S.K.) when they wanted to discontinue participation for any reasons. Weekly follow-up continued until 30 April 2016 (maximum of 24 weeks of follow-up). 

Participants received additional questionnaires on pregnancy outcome and infertility treatment in November 2016 and November 2017. In the 2016 follow-up participants were asked about coital frequency in the past three months or during three-month period preceding pregnancy in case they were currently pregnant. If they were currently pregnant, they were asked delivery due date. If they had delivered a baby, date of delivery and gestational week at birth were asked. Participants were asked whether they had ever visited a medical doctor for consultation, examination, or treatment of infertility since the intake in November 2015. In the 2017 follow-up survey, additional information on delivery, current pregnancy status, and type of infertility treatment received in the past two years was asked. 

### 2.3. Laboratory Analysis

Serum specimens were assayed for AMH at SRL Co. Ltd., Tokyo, Japan, using an AMH Gen II ELISA (Beckman Coulter, Brea, USA). Urine specimens of the subsample women were shipped on dry ice to Okayama University of Science and assayed with an enzyme immunoassay (EIA) for pregnanediol-3-glucuronide (PDG) and estrone-glucuronide (E1G) [30,31,32]. Specific gravity was measured to correct for hydration status [33].

### 2.4. Estimating Day of Ovulation and the Fertile Period

Menstrual cycles were defined as starting on the first day of bleeding and lasting until the day before the next menstrual bleed. Day of ovulation was estimated based on the results from the LH ovulation test kits. A fertile period of each cycle was defined as of eight days, starting from six days prior to the estimated day of ovulation until one day after ovulation. 

### 2.5. Measures of Coital Frequency

We used three measures of coital frequency; a recall estimate of coital frequency in the past three months reported at study intake; the recorded number of days with sexual intercourse in a menstrual cycle during the 24 week follow up; and the recorded number of days with sexual intercourse in a fertile period of a menstrual cycle during the 24 week follow up. 

### 2.6. Statistical Analyses

The primary outcome was spontaneous (natural) conception. The 24-week follow-up data were analyzed as a cycle-level dataset. In cases where participants discontinued follow-up or initiated infertility treatment, succeeding cycles were censored. With the cycle-level dataset, a mixed effects logistic regression model was used to treat individual as a random effect to correct for repeated measures (multiple cycles from each participant). The exposure variables were coital frequency, age (23–28, 29–30, 31–34 years), BMI (15.9–19.4, 19.4–21.5, 21.5–28.9 kg/m^2^), and serum AMH concentrations (0.18–3.17, 3.18–6.13, 6.14–34 ng/mL), which are based on sample tertiles. Three measures of coital frequency were added in each model: recorded coital frequency (number of days with intercourse) in a fertile period (model 1), recorded coital frequency (number of days with intercourse) in a whole cycle (model 2), or reported coital frequency (number of days with intercourse per month) at enrolment (model 3). Odds ratios (ORs) and their 95% confidence intervals (CI) were calculated. With the two-year follow-up data, only descriptive statistics are presented. For the one and two-year follow up data, the likelihood of natural and assisted conceptions are not independent and thus the hazard ratios or odds ratios of any one type of conception cannot be reliably estimated.

All the statistical analyses were done using R ver. 3.3.0 [34].

## 3. Results

### 3.1. Descriptive Results

#### 3.1.1. Participant Characteristics

The mean (SD) age of the participants was 29.5 (2.7) years and the mean BMI was 20.8 (2.4) kg/m^2^ (Table 1). No participants had ever given birth, but 19% (15) of the participants had been pregnant at some point (gravid) by the time of intake. The median (interquartile range) of serum AMH concentration was 5.1 (2.8, 8.0) ng/mL.

#### 3.1.2. Pregnancy Outcomes by 24-Week and Two-Year Follow-Up

A total of 80 women participated in the Baby Machi study among whom 44% (35) spontaneously conceived across 24 weeks. Four participants were lost to follow-up by 24 weeks, among whom three dropped out and one withdrew to start contraceptives per the advice of her doctor. Among the 35 detected pregnancies, 20% (7) ended in fetal loss, 77% (27) resulted in live birth, and the outcome of one pregnancy was unknown. 

By the end of two-year follow-up, 50% (40) had delivered or were currently pregnant by natural conception. Another 24% (19) conceived with assisted reproduction. Four (5%) women did not respond to the final follow-up questionnaire sent out two years after intake and thus their reproductive outcomes were unknown.

#### 3.1.3. Estimated Day of Ovulation, Fertile Period, and Coital Activity

The estimated day of ovulation indicated by the LH kits and the day of luteal transition (DLT) method showed reasonable agreement (Table S1). In 24 weeks, 80 women recorded 319 menstrual cycles with the number of days with positive LH test results ranging from 0 to 5 or higher (Table S2). Among 319 cycles, 73 cycles had zero days with positive LH test results (Table S2). Of the 73 cycles with no positive LH test results, eight cycles resulted in natural conceptions. Of the 246 cycles with one or more days with positive LH test results, 27 cycles resulted in natural conceptions.

**Figure 1:**
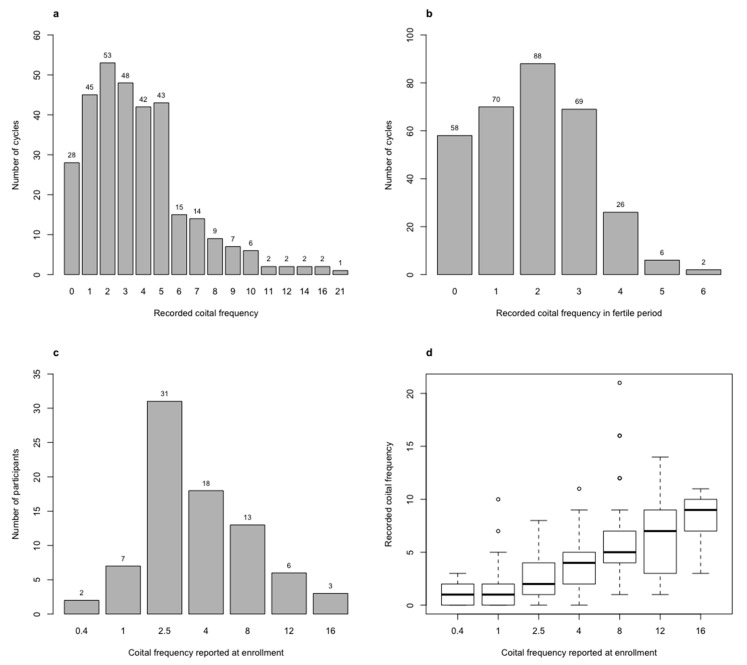
Histogram of (**a**) recorded coital frequency in a cycle, (**b**) recorded coital frequency in a fertile period, (**c**) reported coital frequency per month at enrolment, and (**d**) boxplot of recorded coital frequency in a cycle by reported coital frequency per month at enrolment.

Distributions of coital frequency and the association between recorded and reported coital frequency are shown in Figure 1. In 18% (58/319) of the recorded cycles there was no intercourse in a fertile period (Figure 1b).

### 3.2. Determinants of Probability of Pregnancy

Coital frequency was positively associated with the probability of natural conception in 24 weeks, regardless of coital frequency measure (Table 2). The OR (95% CI) of conception for recorded coital frequency in a fertile period was 1.70 (95% CI 1.05, 2.74) (model 1). It was 1.25 (95% CI 1.04, 1.50) for recorded frequency in a cycle (model 2), and 1.23 (95% CI 1.02, 1.47) for coital frequency reported at enrolment (model 3). None of the individual level factors, i.e., age, BMI, or AMH concentration, were significantly associated with the probability of pregnancy in 24 weeks (Table 2). 

### 3.3. Infertility Treatment

During the first 24-weeks of follow-up, 24% (19) of women consulted a doctor about infertility, among whom nine received guidance on a timing method, six underwent ovulation induction, and three received artificial insemination. 

Within one year after intake, 45% (36) had ever consulted a medical doctor about infertility. Types of treatment (not mutually exclusive) include ovulation induction (*n* = 14), artificial insemination (*n* = 11), and in vitro fertilization (IVF, *n* = 1). Out of the 36 who consulted a medical doctor about infertility within one year, 26 conceived by the end of the two-year follow-up. By the two-year follow-up, 56% (45) had consulted a medical doctor about their fertility issues: at least five received micro-insemination and at least four received IVF.

## 4. Discussion

This prospective pregnancy study of prime reproductive age nulliparous Japanese women revealed that a behavior-related variable—the frequency of intercourse—was associated with an increased likelihood of a conception cycle. Probability of conception in a cycle was not significantly associated with age, BMI, or AMH, a measure of reproductive age and ovarian reserve.

The probability of pregnancy in the present sample, compared to previous studies, was low. The cumulative rate of natural conception was 44% within 24 weeks, which is lower than reported in previous studies, e.g., 60% after six months in Danish couples [35] and 81% after six cycles in German couples [36]. The observed proportion (20%) of detected pregnancies that ended in fetal loss is comparable to the value reported in a previous prospective study on fecundability, where 22% of the 198 pregnancies detected by urinary hCG ended before pregnancy was detected clinically [37].

Coital frequency was low in our sample. The median (interquartile range, IQR) of sexual intercourse was 3 (IQR 2–5) days per cycle in the present sample, which is about half the frequency of six (IQR 4–9) times per cycle in a cohort of US couples trying to conceive [38]. The proportion of cycles with no intercourse in a fertile cycle was as high as 18% in the present sample, which may have contributed to the relatively low cumulative pregnancy rates. The low coital frequency is consistent with the two studies targeting women in Japan [27,39].

Our results show that more frequent intercourse is associated with a higher probability of conception in a cycle. That more coitus will increase the likelihood of pinpointing the fertile period is not unexpected. With low rates of coital frequency, however, more precise identification of the fertile period becomes more critical. A previous study on German couples trying to conceive with natural family planning methods, characterized by self-monitoring of ovulation and timing intercourse, reported a cumulative pregnancy rate as high as 90% after 12 cycles [36], which supports that having well-timed intercourse can increase the probability of conception. Couples in the present study might have been more likely to have coitus in a fertile period than the average Japanese couple trying to conceive, because they were actively monitoring their fertile periods using LH test kits. 

Contrary to our prediction, we found that being slightly underweight was not associated with a lower probability of conception in this sample of Japanese women. Wise et al. [40] reported higher fecundability for underweight parous women but reduced fecundability for underweight nulliparous women. Several studies have reported longer TTP among underweight women compared to normal weight women [21,22,23]. Considering that studies have reported the adverse effects of underweight on the success rate of IVF and intracytoplasmic sperm injection (ICSI) [24,25,41] and that the use of IVF and ICSI are widespread in Japan [3], the potential effect of underweight on the outcome of reproductive technologies should be investigated in future studies.

Neither chronological age nor reproductive age (as measured by AMH) were significantly associated with the probability of conception, after adjusting for frequency of intercourse, in the present sample of reproductive-age women. These results suggest that in this young group of women, ovarian reserve is not a predictor of the probability of natural conception. In previous research, after adjusting for chronological age, a lower AMH concentration was associated with reduced fecundability in one study [29] but not in other [42,43] prospective studies. A cross sectional study reported no significant difference in AMH levels between infertile and fertile women below the age of 40 years [44]. There were only two women with AMH concentrations below 1.1 ng/mL, one of whom naturally conceived during 24-weeks of follow-up. Low AMH values may indicate poor ovarian reserve or premature ovarian insufficiency. High AMH may indicate the presence of PCOS or functional hypogonadotropic hypogonadism [45].

The overall cumulative pregnancy rate in two years was 74% (50% (40) due to natural and 24% (19)) due to assisted conception, in the present study. The use of assisted reproduction by two-year follow up was high in our sample. It is possible that initiation of infertility treatment may alter the probability of natural conception through changes in sexual activity, physiological functions, and other factors. Future research should also investigate how assisted reproduction can affect fecundability and fertility at a population level. 

Limitations of the present study include small size and limited representativeness of the sample. The sample may be biased toward low fecundability, given that these couples had been trying to conceive for several months at intake, while most couples with higher probabilities of pregnancy are expected to conceive within the first few months after discontinuing contraception. We only measured one reproductive biomarker of several that could indicate subfertility and may therefore be missing biological effectors of subfertility. Male factors including semen quality were not examined in the present study. Strengths of the study include the prospective week-by-week follow up of participants, with a low rate of drop-out and detailed behavioral and biological cycle level data.

## 5. Conclusions

Coital frequency and natural conception rates were both low, and higher coital frequency was associated with increased probability of natural conception within 24 weeks, in young Japanese couples trying to conceive their first child. Chronological age, reproductive age as represented by AMH, and BMI were not associated with the probability of pregnancy. Our results suggest that first, natural conception rates could potentially increase with more frequent and well timed intercourse, and second that further work is needed to understand why even in a motivated sample of women monitoring their fertile periods, both the conception and coitus rates were low.

## Figures and Tables

**Table 1:** Individual characteristics of the participants (*n* = 80) in the Baby Machi study by natural conception during 24-weeks follow-up, and any conception (natural or assisted) during 2 year follow up. Median (interquartile range), mean (SD), or *n* (%).

Variable	Total (*n* = 80)	Natural Conception in 24-Weeks		Conception in Two-Years ^3^	
		No (*n* = 45)	Yes (*n* = 35)	No (*n* = 17)	Yes (*n* = 59)
**Observed Number of Menstrual Cycles**	5.0 (2.0, 6.0)	6.0 (5.0, 7.0)	2.0 (1.5, 3.5)	-	-
**Age (Year)**	29.5 (2.7)	29.9 (2.8)	28.9 (2.6)	30.4 (2.1)	29.3 (2.9)
**Partner’s Age (Year)**	31.8 (4.7)	31.9 (5.0)	31.7 (4.4)	32.6 (6.1)	31.1 (3.9)
**BMI (kg/m^2^)**	20.8 (2.4)	21.0 (2.0)	20.4 (2.8)	21.5 (2.4)	20.5 (2.4)
15.9–19.4	27 (34%)	12 (27%)	15 (43%)	3 (18%)	23 (39%)
19.4–21.6	26 (33%)	16 (36%)	10 (29%)	6 (35%)	19 (32%)
21.6–28.9	27 (34%)	17 (38%)	10 (29%)	8 (47%)	17 (29%)
**DUI (Month)**	6 (3, 18)	6 (3, 24)	6 (3, 13)	7 (3, 20)	6 (3, 14)
<12	50 (63%)	25 (56%)	25 (71%)	10 (59%)	38 (64%)
12–23	14 (18%)	5 (11%)	9 (26%)	3 (18%)	11 (19%)
24+	11 (14%)	10 (22%)	1 (3%)	3 (18%)	6 (10%)
Do not remember	5 (6%)	5 (11%)	0 (0%)	1 (6%)	4 (7%)
**Smoking**					
Never	67 (84%)	39 (87%)	28 (80%)	14 (82%)	50 (85%)
Quit	9 (11%)	4 (9%)	5 (14%)	2 (12%)	7 (12%)
Current	4 (5%)	2 (4%)	2 (6%)	1 (6%)	2 (3%)
**Partner’s Smoking**					
Never	48 (60%)	27 (60%)	21 (60%)	12 (71%)	34 (58%)
Quit	15 (19%)	7 (16%)	8 (23%)	2 (12%)	12 (20%)
Current	17 (21%)	11 (24%)	6 (17%)	3 (18%)	13 (22%)
**Gravidity**					
Gravid	15 (19%)	8 (18%)	7 (20%)	3 (18%)	10 (17%)
Nulligravid ^1^	65 (81%)	37 (82%)	28(80%)	14 (82%)	49 (83%)
**Serum AMH (ng/mL)**	5.1 (2.8, 8.0)	5.6 (2.7, 8.6)	4.4 (2.8, 6.8)	5.8 (4.1, 9.3)	4.8 (2.9, 7.7)
d**Coital Frequency ^2^**	3.3 (2.5, 8.0)	2.5 (2.5, 4.0)	4.0 (2.5, 8.0)	4.0 (2.5, 8.0)	4.0 (2.5, 8.0)

AMH, antimüllerian hormone. DUI, duration of unprotected intercourse at enrolment. ^1^ Includes *n* = 1 participant who reported that she did not know her gravidity status. ^2^ Per month number of days with sexual intercourse in the past three months, reported at intake. ^3^ *n* = 4 women were lost to follow-up.

**Table 2:** Odds ratios and 95% confidence intervals for natural conception in 24-weeks of sample women from mixed effect logistic regression models (*n* = 319 cycles from *n* = 80 women).

Predictors	Model 1	Model 2	Model 3
**Individual Factors**			
Age (Ref.: 23–28)			
29–30	0.34 (0.06, 1.93)	0.25 (0.05, 1.32)	0.26 (0.05, 1.25)
31–34	0.23 (0.04, 1.34)	0.26 (0.05, 1.24)	0.26 (0.06, 1.13)
BMI (Ref.:19.4–21.5)			
15.9–19.4	3.40 (0.54, 21.3)	2.61 (0.51, 13.4)	2.46 (0.53, 11.3)
21.5–28.9	0.72 (0.13, 3.96)	0.64 (0.13, 3.0)	0.58 (0.14, 2.47)
AMH, 0.18–3.17 ng/mL			
3.18 to 6.13 ng/mL	0.81 (0.16, 4.08)	0.59 (0.13, 2.59)	0.47 (0.11, 1.98)
6.14 to 34 ng/mL	0.40 (0.08, 2.10)	0.27 (0.05, 1.34)	0.28 (0.06, 1.22)
Coital frequency reported at enrollment	-	-	1.23 (1.02, 1.47)
**Cycle Factors**			
Recorded coital frequency in fertile period ^†^	1.70 (1.05, 2.74)	-	-
Recorded coital frequency in a cycle	-	1.25 (1.04, 1.50)	-

^†^ A fertile period of each cycle was defined as eight days, starting from six days prior to the estimated day of ovulation until one day after ovulation.

## Supplementary Materials

The following are available online at https://www.mdpi.com/1660-4601/17/14/4985/s1, Figure S1: An example (*n* = 1 woman with multiple cycles) of specific gravity (SG)-corrected daily concentrations of estrone-glucuronide (E1G) and pregnanediol-glucuronide (PDG) in urine with daily information on menstrual bleed, sexual intercourse, and ovulation (based on urinary LH) and pregnancy (based on urinary hCG) test results, Table S1: Difference in days between day of luteal transition (DLT) and the first day of positive ovulation test in each cycle with DLT and at least 1 day with positive ovulation tests (*n* = 52 cycles), Table S2: Number of menstrual cycles by number of days with positive LH test results and spontaneous conception during 24-weeks follow-up.
