# Supplementary material for: Coital Frequency and the Probability of Pregnancy in Couples Trying to Conceive Their First Child: A Prospective Cohort Study in Japan
Source: Int J Environ Res Public Health. 2020 Jul 10;17(14):4985. doi: 10.3390/ijerph17144985 (PMC7399901; doi:10.3390/ijerph17144985)
Supplement: Supplementary file 1 [file ijerph-17-04985-s001.pdf]

Supplementary Materials: Coital Frequency and the Probability of Pregnancy in Couples Trying to Conceive their First Child: A Prospective Cohort Study in Japan

Shoko Konishi, Tomoko T. Saotome, Keiko Shimizu, Mari S. Oba and Kathleen A O'Connor

Recruitment was performed online in two time periods: from October 7 to 21 and from November 6 to 8, 2015. Potential participants were recruited mainly via advertisements on the menstrual calendar app Lalune (Ateam Inc., Japan) and also via posts on Facebook and Twitter and emails. Interested individuals were invited to view the website of the Baby Machi study, which described study objectives and procedures in detail. Eligibility was checked using a self-administered questionnaire online, and only eligible women could enroll in the present study. There were 11,614 views of the Baby Machi study website that described study objectives and protocol. A total of 152 women who met the eligibility criteria enrolled in the study, among whom 80 women participated in the intake session at the University of Tokyo. The participants used a home pregnancy test (Dotest hCG, ROHTO Pharmaceutical Co., Ltd., Japan) on the morning of study intake (November 15, 2015). Only those women who obtained negative results were eligible.

In a baseline questionnaire participants were asked to select one answer from 10 categories that match their coital frequency in the past three months: five days or more per week, four days per week, three days per week, two days per week, one day per week, two or three days per month, one day per month, one day per two or three months, less than one day per two to three months, or do not know/do not want to answer. For statistical analysis, the coital frequency categories were converted into a continuous variable number of days with intercourse per month. The timing and method of the last contraceptive use were asked. The duration of unprotected intercourse (DUI) at intake was calculated as the number of months between last contraceptive use and study intake.

During 24-weeks follow-up, day of sexual intercourse was reported in the weekly diary, separately for intercourse with or without intravaginal ejaculation. In the present paper the frequency of sexual intercourse with intravaginal ejaculation was used for analysis. In the weekly diary, participants were also asked whether they visited a doctor (either for pregnancy tests or for infertility treatment) during the past week. If they answered "yes", then they were asked about the diagnosis for pregnancy or type of infertility treatment they received. If the participants were diagnosed as being (likely to be) pregnant by a medical doctor, an additional questionnaire was sent four weeks later to enquire about the status of the pregnancy.

Menstrual bleeding was defined as two or more days of consecutive bleeding. Cycle length was defined as the number of days between the first day of menstrual bleeding and the previous day of the first day of next menstrual bleeding. For cycles having one or more days of a positive ovulation test, one day after the first day of a positive result was assumed to be the day of ovulation. For cycles with no positive LH ovulation test results (73 out of 319 cycles examined), 14 days before the onset of the next menstrual bleed or the 15th day of the menstrual cycle (in cases where the next menstrual cycle onset was unknown) was assumed to be the day of ovulation.

For the subsample of women collecting daily urine specimens for the laboratory measurement of estrogen and progesterone metabolites, the ratio of daily urinary concentrations of E1G and PDG was used to estimate the day of ovulation using the day of luteal transition (DLT) method [1] and compared with the day of ovulation estimated by the LH ovulation test kits (Dotest hCG, ROHTO Pharmaceutical Co., Ltd., Japan). The estimated day of ovulation, using DLT vs. the LH ovulation test, was compared using information on menstrual cycles with at least one day of positive LH test and an estimated day of ovulation based on the DLT method. For comparison, differentials (in days) between estimated days of ovulation based on both methods were computed and tabulated.

Pregnancy in this study was defined in two ways. For the 24-weeks follow-up, if the gestational sac was confirmed by a medical doctor and the conception was achieved without any infertility treatment, it was defined as a natural (or spontaneous) conception. These conceptions either ended in live birth or spontaneous abortions.

For the two-year follow-up, any ongoing pregnancies or pregnancies that ended in live births were treated as pregnancies, which include both natural (spontaneous) and assisted conceptions. Pregnancies that ended in abortions before the time of survey were not considered in this analysis.

An example of a daily urinary hormone profile and pregnancy and ovulation test results are shown in Supplemental Figure 1. Eighteen women provided daily urine specimens for 87 cycles. Using the DLT method, day of ovulation could not be determined for 20 cycles either due to censored cycles (n = 17) or unclear hormonal profile (n = 3). Day of ovulation could be determined for the rest of 67 cycles out of which 15 cycles had no LH positive results. Of the menstrual cycles (n = 52) with at least one day with LH positive results and day of ovulation determined by the DLT method, the estimated day of ovulation for both methods were on the same day (29%), or the first day of ovulation by the LH kit was one day before the DLT (38%) (Table S1). In 43 out of 52 cycles (83%), the differences between the first positive LH day and the DLT day were within plus or minus two days (Table S1) and thus showed reasonable agreement. Most of the cycles (88%) had either two, one, or zero days with positive LH test results (Table S2). The proportion of conception cycles were 11%, 9%, and 11% respectively, for cycles with zero, one, or two days with positive LH test results (Table S2).

Table S1. Difference in days between day of luteal transition (DLT) and the first day of positive ovulation test in each cycle with DLT and at least 1 day with positive ovulation tests (n = 52 cycles).

Days Between the First Day of LH+ to DLT	Number (Proportion) of Cycles
−17	1 (2%)
−4	1 (2%)
−2	2 (4%)
−1	3 (6%)
0	15 (29%)
1	20 (38%)
2	3 (6%)
3	1 (2%)
4	3 (6%)
6	1 (2%)
14	1 (2%)
28	1 (2%)
Total	52 (100%)

DLT, day of luteal transition.

Table S2. Number of menstrual cycles by number of days with positive LH test results and spontaneous conception during 24-weeks follow-up.

Number of Days with Positive LH Test Results	Total	Conception		Proportion of Cycles with Conception
		No	Yes	
0	73	65	8	11%
1	102	93	9	9%
2	106	94	12	11%
3	30	26	4	13%

4	6	5	1	17%
5+	2	1	1	50%
Total	319	284	35	11%

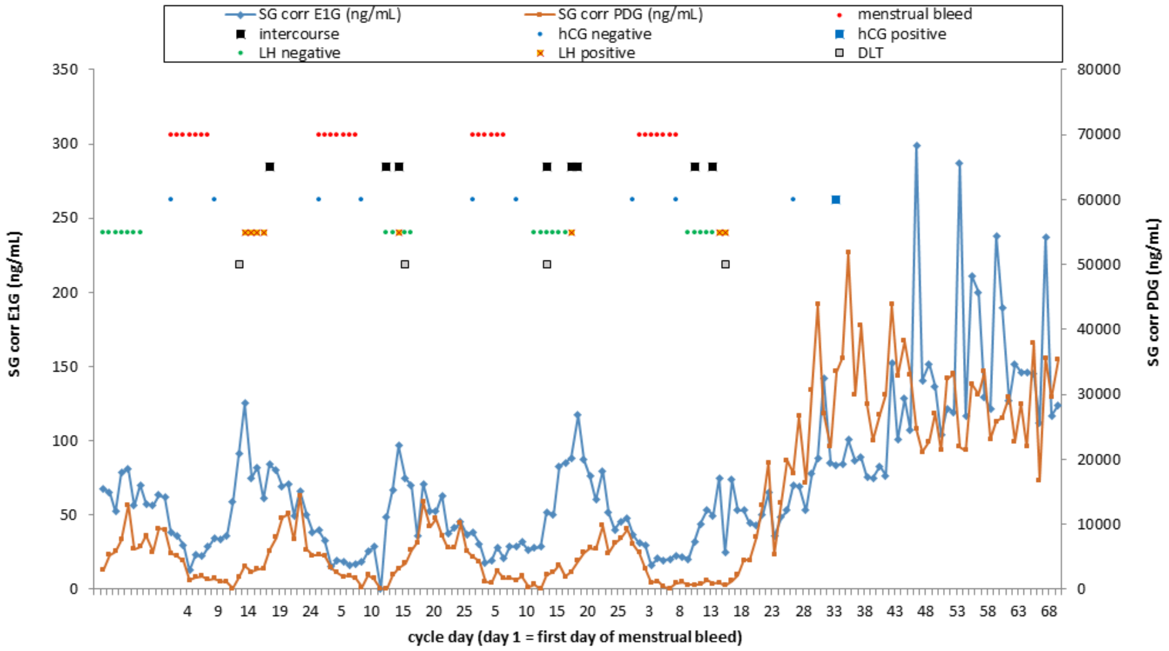

Figure S1. An example (*n* = 1 woman with multiple cycles) of specific gravity (SG)-corrected daily concentrations of estrone-glucuronide (E1G) and pregnanediol-glucuronide (PDG) in urine with daily information on menstrual bleed, sexual intercourse, and ovulation (based on urinary LH) and pregnancy (based on urinary hCG) test results. DLT indicates the estimated day of luteal transition [1].

References

1. Baird, D.D.; Weinberg, C.R.; Wilcox, A.J.; McConnaughey, D.R.; Musey, P.I. Using the ratio of urinary oestrogen and progesterone metabolites to estimate day of ovulation. *Stat. Med.* **1991**, *10*, 255–266.
2.

© 2020 by the authors. Submitted for possible open access publication under the terms and conditions of the Creative Commons Attribution (CC BY) license (http://creativecommons.org/licenses/by/4.0/).
